# Light-Driven Linear Inchworm Motor Based on Liquid Crystal Elastomer Actuators Fabricated with Rubbing Overwriting

**DOI:** 10.3390/ma14216688

**Published:** 2021-11-06

**Authors:** Mikołaj Rogóż, Jakub Haberko, Piotr Wasylczyk

**Affiliations:** 1 Photonic Nanostructure Facility, Faculty of Physics, University of Warsaw, ul. Pasteura 5, 02-093 Warsaw, Poland; pwasylcz@fuw.edu.pl; 2 Faculty of Physics and Applied Computer Science, AGH University of Science and Technology, Al. Mickiewicza 30, 30-059 Kraków, Poland; haberko@fis.agh.edu.pl

**Keywords:** liquid crystal elastomer, linear motor, actuator, smart material, rubbing

## Abstract

Linear displacement is used for positioning and scanning, e.g., in robotics at different scales or in scientific instrumentation. Most linear motors are either powered by rotary drives or are driven directly by pressure, electromagnetic forces or a shape change in a medium, such as piezoelectrics or shape-memory materials. Here, we present a centimeter-scale light-powered linear inchworm motor, driven by two liquid crystal elastomer (LCE) accordion-like actuators. The rubbing overwriting technique was used to fabricate the LCE actuators, made of elastomer film with patterned alignment. In the linear motor, a scanned green laser beam induces a sequence of travelling deformations in a pair of actuators that move a gripper, which couples to a shaft via friction moving it with an average speed in the order of millimeters per second. The prototype linear motor demonstrates how LCE light-driven actuators with a limited stroke can be used to drive more complex mechanisms, where large displacements can be achieved, defined only by the technical constrains (the shaft length in our case), and not by the limited strain of the material. Inchworm motors driven by LCE actuators may be scaled down to sub-millimeter size and can be used in applications where remote control and power supply with light, either delivered in free space beams or via fibers, is an advantage.

## 1. Introduction

Nature does not seem to use rotary motion often. We are aware of only three examples of rotary motion used in the animal kingdom: bacterial flagella are powered by a rotating motor built into the cell envelope, driven by the flow of protons or sodium ions; dung beetles use rolling to transport loads; and some caterpillars from the *Crambidae* family roll away when in danger.

Our civilization, on the contrary, relies chiefly on rotary motion for energy conversion, transport and manufacturing. At the same time, linear motion is vital for many applications, e.g., in handling, positioning and metrology. There are a number of solutions for realizing linear displacement with rotary motors: via rack and pinion, wheel and belt or lead screw. It is also possible to generate linear displacement directly with either a pressurized medium acting on a piston, as in hydraulic and pneumatic actuators, or electromagnetic forces, as in voice coils and more complex linear electric motors. Another approach is based on direct material deformation, most notably in piezoelectric actuators, or in more exotic examples, based on shape memory alloys, or other shape memory material, such as liquid crystalline epoxy resins and composites [1,2], dielectric elastomers or liquid crystal networks [3,4]. Each of these solutions has certain advantages and limitations and several characteristics are taken into account when considering specific applications, among them: speed; stroke; maximum load that can be handled; maximum force that can be generated; accuracy; and locking force at rest with no power supply. In some applications, such as positioners for nuclear magnetic resonance (NMR) scanners, the materials involved in the actuator constructions can be critical (e.g., non-metallic or non-magnetic components only), while in others, e.g., in environments sensitive to electromagnetic interference, remote control without electric currents may be required.

Liquid crystal elastomers (LCEs) are elastic polymers with an ability to reversibly deform under external stimuli, e.g., temperature. Their rod-shaped molecules can be aligned in cross-linked polymer chains and, upon this arrangement, the order (anisotropic)–disorder (isotropic) transition reduces the effective length of these chains along the alignment direction (the director). This is accompanied by an increase in the spacing between the molecules in perpendicular directions. Consequently, the material can exhibit large, fast and reversible shape changes; thus created deformation geometry is determined by the molecular alignment within the material [5] and can generate forces well exceeding the weight of the deforming element [6,7]. The mechanisms responsible for the photomechanical response can be either photochemical reactions, often associated with the cis–trans isomerization, or photothermal heating, when the temperature of the material (locally) increases due to light absorption, which can be improved by adding a suitable dye or other absorber. In the case of such heating, generated by light absorption, the fast photomechanical response can be used to remotely power and control mechanisms [8,9,10]. Selective heating by temporally and/or spatially modulated laser beam(s) has been demonstrated in devices performing various tasks, including rotary micro-motors [11], also in micro-scale [12,13,14].

Inchworm motors were initially developed as rotary mechanisms with a rotor moved by a sequential action of piezo actuators [15,16]. In a linear inchworm motor, a number of actuators are operated in a sequence, consisting of gripping, moving and releasing the shaft. Inchworm motors in various forms, most commonly with piezoelectric actuators and millimeter- to centimeter-scale are now widely used, mainly in precision fabrication and positioning. Their most prominent advantages are: infinite motion (i.e., not limited by the step length); high speed; sub-micrometer precision and accuracy; high locking force also with no power supply. At the same time, if driven with piezo actuators, inchworm motors require high voltages, typically in the order of tens to hundreds of volts, and dedicated controllers for reliable operation.

Here, we present a light-driven linear motor, using the inchworm principle, with two accordion-like LCE actuators. The actuators use crosslinked liquid crystal elastomer film, polymerized with UV light, with red dye added to increase green light absorption [5]. Photothermal response in the 50 micron LCE film with patterned molecular alignment, fabricated by the rubbing overwriting technique, is used to convert light energy into the movement of a carbon fiber shaft. A spatially modulated laser beam creates a traveling deformation along the actuators that displace a gripper, which is friction-coupled to the shaft. Bi-directional operation is possible with reversed spatiotemporal modulation (scanning) of the laser beam.

## 2. Materials and Methods

### 2.1. Light Responsive Actuators

**Figure 1:**
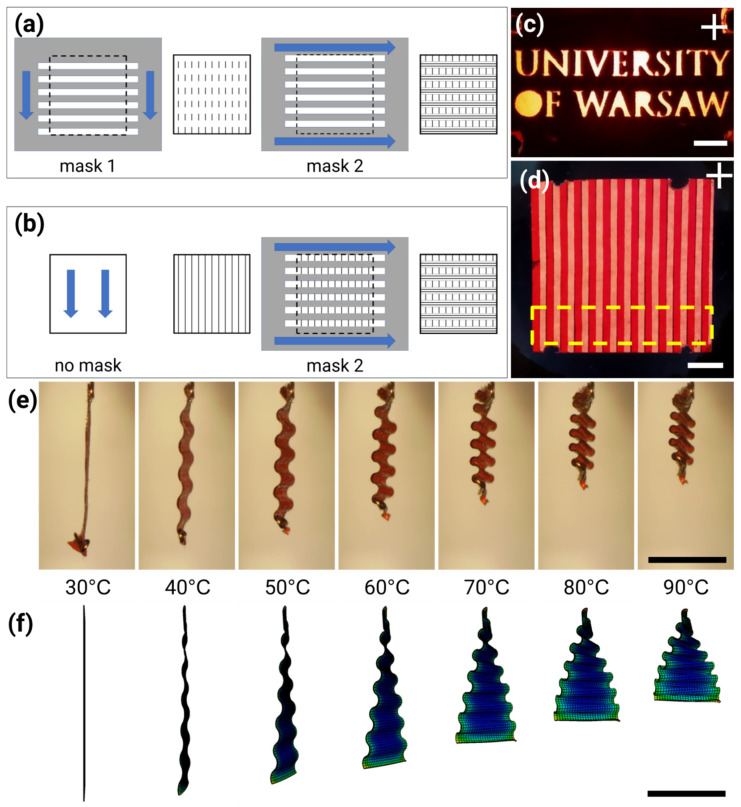
Two approaches to the fabrication of LCE films with patterned orientation. In the previously used method, two masks (grey) were used, one for each rubbing direction (indicated by blue arrows and grey lines) (**a**). In the rubbing overwriting method, the entire surface is rubbed in one direction first and one mask is used for the regions with a perpendicular rubbing direction (**b**). (**c**,**d**) LCE films prepared with rubbing overwriting technique using: (**c**) a mask with the University of Warsaw logo cut out and (**d**) a mask with 1 mm wide slits cut out. The yellow box shows how the accordion-like actuator was cut from the film. White light photographs were taken between crossed polarizers, with the white cross showing the orientation of the polarizers. (**e**) Accordion-like deformation in a stripe of LCE film cut perpendicular to the pattern lines, heated from 30 °C to 90 °C. (**f**) Finite element simulation of the LCE stripe deformation. Temperature values relate to conditions in (**e**,**f**). All scale bars are 5 mm long.

To fabricate light-responsive actuators made of LCE films with patterned molecular alignment, we introduced the technique of mechanical rubbing overwriting. There are several techniques used for inducing molecular orientation in liquid crystals, as well as in the fabrication of liquid crystal polymers or networks, and some of them allow for patterned orientation: laser written gratings [17,18]; photo-alignment [19,20]; imprint lithography [21]; and micro-rubbing [22]. Mechanical rubbing is widely used for liquid crystal orientation: after coating a glass surface with a thin layer of soft material, typically a polymer, a velvet-like cloth is rubbed in one direction. This induces a preferential direction of the molecular orientation in the liquid phase, once such prepared glass is used to make a thin cell, infiltrated with the liquid crystal. The mechanisms involved include micro-grooves being formed and/or local heating of the coating material above the melting point and modification of the surface, as the solidification front moves along, so that the LC molecules anchor to such treated surface, oriented along the rubbing direction [23]. Rubbing is often used to fabricate LCE films with simple orientations, uniform across the whole film area such as nematic [24,25,26] or twisted nematic [27,28]. We demonstrated how rubbing with masks that leave only some fragments of the surface exposed could be used to prepare LCE films with patterned orientations. Films with different areas having different molecular alignment and, as a result, different photomechanical responses (deformations) to a stimulus—temperature or light—were fabricated and used to demonstrate a light-driven crawling caterpillar micro-robot [29]. This procedure involved the mechanical rubbing of glass slides spin-coated with a layer of poly (vinyl alcohol) with a home-built rotating drum rubbing machine and two masks (hand- or laser-cut out of paper), one for each orientation of the rubbing direction (Figure 1a).

In [30], it was reported that a homogeneous substrate rubbed in two different directions results in the alignment of liquid crystals along the axis oriented between these two directions. The dependence of the alignment axis angle on the number of strokes in the first and second (perpendicular) rubbing directions was presented, together with a theoretical model. Authors of [31] described a method involving the unidirectional rubbing of a polyimide coating, which was subsequently partly covered with a mask formed by photolithography. The surface was then rubbed perpendicular to the first rubbing direction and the mask was removed. The rubbed coating thus produced induced position-dependent orientation of molecules in a liquid crystal light modulator.

Inspired by these results, we have introduced a new approach to producing patterned rubbing—which we call rubbing overwriting—where we combine unidirectional and multidirectional rubbing by applying a mask. In the first step, the entire surface is exposed and rubbed in one direction, and in the second step the mask covers certain areas and the exposed surface is rubbed in the perpendicular direction (Figure 1b). In this new procedure, the tedious step of mask alignment is eliminated.

A 50 µm-thick cell was made with two glass slides oriented in such a way that the areas were rubbed in a certain direction on one slide face the areas rubbed in perpendicular direction on the other slide. The cell was filled with the monomer mixture containing 83 mol% of monomer 4-methoxybenzoic acid 4-(6-acryloyloxy-hexyloxy) phenyl ester (Synthon Chemicals), 15 mol% of crosslinker 1,4-Bis-[4-(3-acryloyloxypropyloxy)benzoyloxy]-2-methylbenzene (Synthon Chemicals), 1 mol% of photoinitiator 2-benzyl-2-(dimethylamino)-4′-morpholinobutyrophenone (Irgacure 369, Sigma-Aldrich) and 1 mol% of red dye N-ethyl-N-(2-hydroxyethyl)-4-(4-nitrophenylazo)aniline (Disperse Red 1, Sigma-Aldrich). The cell was filled on a hot plate at 80 °C by capillary forces, then heated to 140 °C for 2 min and cooled on an aluminum plate at 22 °C. After 15 min, the mixture was polymerized with a 379 nm UV LED. White light polarizing microscope observations confirmed that the film had rectangular areas with twisted nematic director orientation, where the adjacent segments had their orientation rotated by 90° (Figure 1d) and a stripe cut perpendicular to these segments deformed into the accordion-like shape when heated up (Figure 1e), similar to the results of previously reported experiments, where photo orientation was used for patterned alignment [32].

The homogenous film, using the same LCE composition and heated uniformly in an oven, had the maximum contraction of 35% at 130 °C. The linear actuator stroke could be increased (amplified) using bending instead of contraction and the accordion-like deformation was demonstrated to provide up to an 80% reduction in the initial length of the actuator [29].

### 2.2. Linear Inchworm Motor

**Figure 2:**
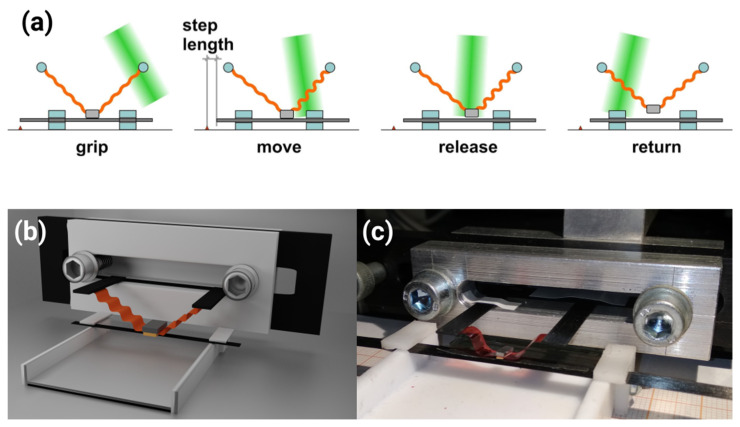
(**a**) The light-driven linear inchworm motor principle of operation. During the slow scan of the laser beam (green), the deformation traveling along two light-responsive elastomer accordion-like actuators (orange) results in the gripper (gray) coupling to the shaft by friction, pulling the shaft by one step, releasing the gripper by pulling it up and returning it to the initial position with the relaxation of the elastomer. Three-dimensional model of the linear motor (**b**) and a photograph of the device (**c**) (the M6 screw heads, 10 mm in diameter, give a scale reference).

Light-induced travelling deformation in an LCE ring was used to demonstrate a rotary micro-motor (5.5 mm in diameter), similar in design to the travelling wave piezoelectric motors used in, e.g., focusing mechanisms of camera lenses [6]. Building on these results, we have designed a light-driven linear motor with infinite stroke, i.e., not limited by the actuator’s deformation, but, in principle, only by the shaft length. By sequential action of the two accordion-like LCE actuators, powered by a scanned laser beam, a small mass (gripper) moves in an orbital trajectory. At the lower part of this trajectory it comes into friction coupling with a shaft and pulls it a step (typically around 1 mm long) in one direction. Repeated cycles of this motion result in large linear displacement of the shaft and the sequence order (defined by the laser beam scanning direction) defines the shaft movement direction (Figure 2a).

Each of the LCE actuators was 13 mm long, 5 mm-wide stripe, cut perpendicular to the segmented rubbing (Figure 2c). One end of the actuator was attached (glued) to a stationary support and the other to a gripper, made of a lead block (106 mg weight) with a piece of 2000 grit sandpaper glued to the bottom surface. A strip cut from the same sandpaper was also glued to the top side of the carbon fiber shaft (0.2 × 5 mm cross section, 120 mm length, 144 mg total weight). The shaft was sliding in two polytetrafluoroethylene (Teflon) slits (Figure 2c). The motor was powered by a laser beam (shaped with a cylindrical lens into an ellipse with full widths at half maximum of 4.5 mm and 0.4 mm), reflected from a mirror mounted on a galvo scanner, scanned along the actuators. The galvo scanner was controlled by an asymmetric sawtooth signal from a waveform generator. Each slow scan of the laser beam across the two LCE actuators (scanning speed on the actuator surface was between 21 mm/s for 0.4 Hz stepping frequency and 47 mm/s for 0.9 Hz stepping frequency) resulted in one motor step, consisting of: gripping the shaft; moving (pulling) the shaft by the gripper; detaching the gripper from the shaft; and returning the gripper to the initial position, as presented in Figure 2a. The slow scanning direction determined the direction of the net shaft movement. The fast backward scan of the laser beam (scanning speed on the actuator surface above 3 m/s) did not induce any visible response in the LCE actuators [33]. The motor operation was recorded with a CMOS camera (640 × 480 pixels, 90 fps, DLT-Cam PRO 5 MP, Delta Optical, Nowe Osiny, Poland) mounted on a stereo microscope. To visualize the thermal response of the LCE actuators after light absorption, an infrared camera was also used (320 × 240 pixels, 60 fps, FLIR A35sc, FLIR, Wilsonville, OR, USA).

The photomechanical response of the LCE actuator was simulated using the finite element method (FEM) with coupled temperature-displacement implicit analysis (SIMULIA, Abaqus/CAE). The actuator was modeled as a 13 mm × 5 mm × 50 μm cuboid and the LCE as an elastic material with a 500 MPa Young’s modulus and 0.4 Poisson’s ratio. The contraction of the polymer network in response to a thermal stimulus was taken into account as a negative temperature expansion coefficient along the director orientation (α_33_) and a positive coefficient in two other orthogonal directions (α_11_, α_22_). The total sample volume was made constant by setting α_11_ = α_22_ = −0.5·α_33_. Based on the experimental results, the contraction was modeled to take place within the 30–120 °C temperature range and was up to 30%. Mass density, specific heat and thermal conductivity were 960 kg m^−3^, 1200 J kg^−1^ K^−1^ and 0.49 J m^−1^ K^−1^, respectively. The simulation domain was divided into 1 mm-wide sections along the longest dimension. In each section, the director orientation gradually changed by 90° from the top to the bottom of the actuator; in the neighboring sections, the director arrangement was flipped by 90° [29].

The LCE stripe exchanged heat with the environment by radiation (the emissivity was 0.67) and convection (with a moderate heat transfer coefficient of 0.49 W m^−2^ K^−1^). The temperature was first increased to 120 °C, inducing the wave-like deformation of the actuator, and then decreased to 24.5 °C, resulting in its return to the initial flat state. The experimental results (Figure 1e and Video S1, Supplementary Materials) were compared with the simulation results (Figure 1f and Video S2).

## 3. Results and Discussion

**Figure 3:**
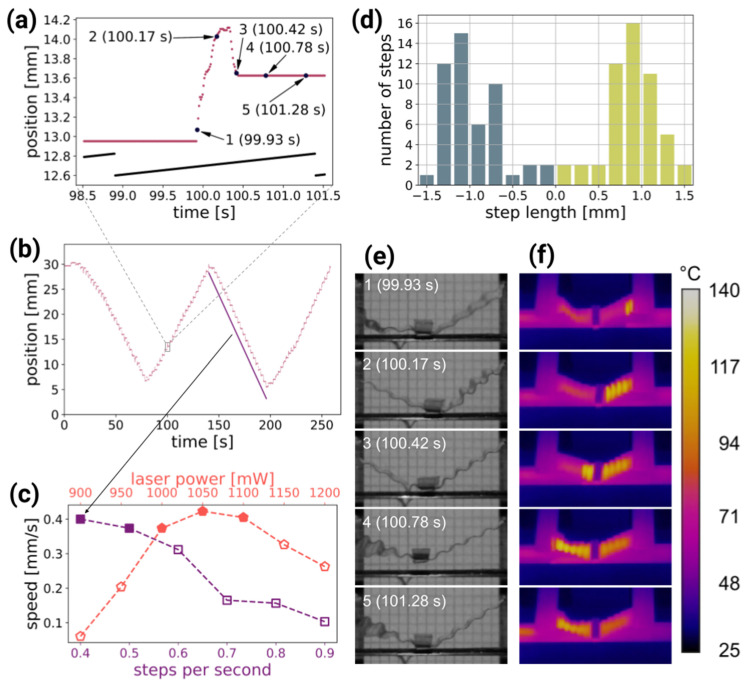
Performance of the light-driven linear inchworm motor: (**a**) horizontal position of the shaft during one laser scanning cycle (red points) and the laser beam scanning galvo signal (black line). The numbers correspond to the positions in the photographs in (**e**). (**b**) The shaft position versus time for continuous operation of the motor, with the scanning direction changed three times. The average shaft speed is measured by fitting straight lines (purple line) to the position over many steps. (**c**) The motor average speed for different scan frequencies and laser powers. Filled symbols represent the parameters where repeatable, linear motor operation was achieved, while open symbols represent parameters where stable operation was not possible (see text for details). (**d**) Step length histogram of the measurement presented in (**c**). The motor operation during one cycle recorded with visible (**e**) and infrared (**f**) camera. A triangular marker is visible, attached at the bottom of the shaft, that was used for position tracking. Scan parameters in (**a**–**e**): 0.4 steps per second, 1 W laser power.

Figure 3 and Video S3 demonstrate the bi-directional operation of the light-driven linear motor. Using image tracking software, the shaft position versus time was measured. The analysis of a single step revealed that the shaft moved forward and then retracted slightly during each cycle of the laser beam scanning (Figure 3a). First, at rest, the gripper was in contact with the shaft, due to gravity and the elasticity of the actuators pushing it towards the shaft. As the first actuator was activated with light, the shaft was pulled by the friction force between the gripper and the shaft. Just before the gripper detached, being pulled away (in the upward direction) from the shaft, it moved in the opposite direction (Figure 3e,f, frame 100.42 s): the net length of one motor step depends on the difference between these two displacements. In the first few steps in Video S3, the warm-up phase is visible, when the average temperature of the LCE actuators gradually increased with each laser scan, until it reached a steady-state operation. Figure 3f and Video S4, recorded in the infrared, reveal the temperature distribution in the LCE actuators during the motor operation. The actuators were locally heated up by the absorbed laser light, reaching approximately 120 °C, and the photomechanical response, resulting in contraction, followed. This sequence of light absorption → temperature increase → deformation → relaxation depends on the interplay of the properties of the photo-responsive material (absorption coefficient, thermal conductivity) and its coupling to the environment, leading to heat dissipation via conductivity, emission and convection, and has been previously analyzed [33].

To determine the average motor speed, linear functions were fitted to the points corresponding to the unidirectional motor motion (Figure 3b), and the average of the absolute values of the slopes was calculated. The average speed was measured against two parameters: the laser power and the laser beam scanning frequency. The first set of measurements was performed with a constant laser beam scanning frequency (0.5 scans s^−1^) and varying laser power (Figure 3c, red pentagons), and the second with a fixed laser power (1000 mW) and varying scan frequency (Figure 3c, purple squares).

Laser power that enabled motor operation with a scanning frequency of 0.5 scans per second ranged from 900 to 1200 mW. For laser power below this range, the gripper did not release the shaft, and for power above this range, the LCE actuators were damaged. With a constant scanning frequency, the highest average shaft speed (0.42 mm s^−1^) was measured with a laser power of 1050 mW. When using lower powers, the backward shift in each step became longer and the net step length decreased, which resulted in lower average speed. When using higher powers, the LCE actuators absorbed too much energy to be able to relax between scans, which resulted in the shortening of the shaft movement phase within the cycle.

In the second series of experiments, the scanning frequency was changed from 0.4 to 0.9 scans s^−1^ with 0.1 scans s^−1^ step, with a constant laser power of 1000 mW. Overall, the lower the scanning (stepping) frequency, the higher the average shaft speed, with the maximum of 0.40 mm s^−1^ for 0.4 scans s^−1^. For this frequency, the step length histogram is plotted in Figure 3d. It reveals an average step length of (0.94 ± 0.32) mm, with (0.91 ± 0.32) mm and (0.97 ± 0.33) mm for the right and left motor operation directions, respectively.

When using parameters that resulted in a lower shaft speed (below approximately 0.35 mm s^−1^, open symbols in Figure 3c), the motor operation became less stable: the step length varied from scan to scan, and the average shaft speeds depended on the scan direction. In this “non-linear” mode of operation, the motor can still be used, but for accurate positioning, some kind of position sensor and a feedback loop would have to be added.

For some applications, the holding force on the shaft with the unpowered motor is an important parameter. In our linear motor, this force was measured to be 1.3 mN, independent of the direction.

## 4. Conclusions

We used the rubbing overwriting technique for fabricating patterned liquid crystal elastomer films. The method involved multidirectional mechanical rubbing with a mask to obtain materials with complex molecular order. Using the actuators made by this technique, we demonstrated that the idea of a linear inchworm motor can be implemented in a light-powered, centimeter-scale device, using LCE actuators with a thermally mediated photomechanical response. With properly chosen driving parameters—laser power and stepping frequency—reliable operation of the linear motor was achieved.

Although, in the current prototype, the actuator response to the light stimulus is highly nonlinear and the shaft positioning accuracy is limited, the linear motor can still be used in applications where a large stroke is more important than the positioning accuracy or repeatability. For more demanding applications, a contactless position sensor (encoder), e.g., optical or magnetic, can be added, and the motor can operate in the closed loop configuration, where the exact length of each step (or even residual backward steps) is not critical and a wider range of driving parameters can be used. Scanned laser beam illumination can be replaced with a different modulated illumination, e.g., two light sources, activating the entire LCE actuator, operating in sequence.

For the increased precision that may be required in certain applications, the motor can be operated in a hybrid mode, where the stepping action is used to approximately reach the destination position and a proportional response of the actuators is used to fine-tune the final shaft position to within one step length.

The idea of a light-powered inchworm motor can be directly implemented in an infinite-angle-of-rotation rotary motor (positioner) by replacing the straight shaft with a circular one.

With direct laser writing, the LCE actuators can be scaled down to the sub-millimeter scale, where patterned rubbing can—be realized by either photo-alignment [34] or multiple alignment [18].

## Supplementary Materials

The following are available online at https://www.mdpi.com/article/10.3390/ma14216688/s1, Video S1: Thermo-mechanical response of the accordion-like LCE actuator heated in an oven, Video S2: Results of the FEM simulations of the thermo-mechanical response of the LCE actuator, Video S3: Light-driven linear inchworm motor operation, Video S4: Light-driven linear inchworm motor operation filmed in the infrared to visualize the temperature distributions in the actuators.
